# Supplementary material for: Differentiation of Adipose-Derived Stem Cells into Vascular Smooth Muscle Cells for Tissue Engineering Applications
Source: Biomedicines. 2021 Jul 9;9(7):797. doi: 10.3390/biomedicines9070797 (PMC8301460; doi:10.3390/biomedicines9070797)
Supplement: Supplementary file 1 [file biomedicines-09-00797-s001.zip › biomedicines-1250292-supplementary.pdf]

Supplementary Materials

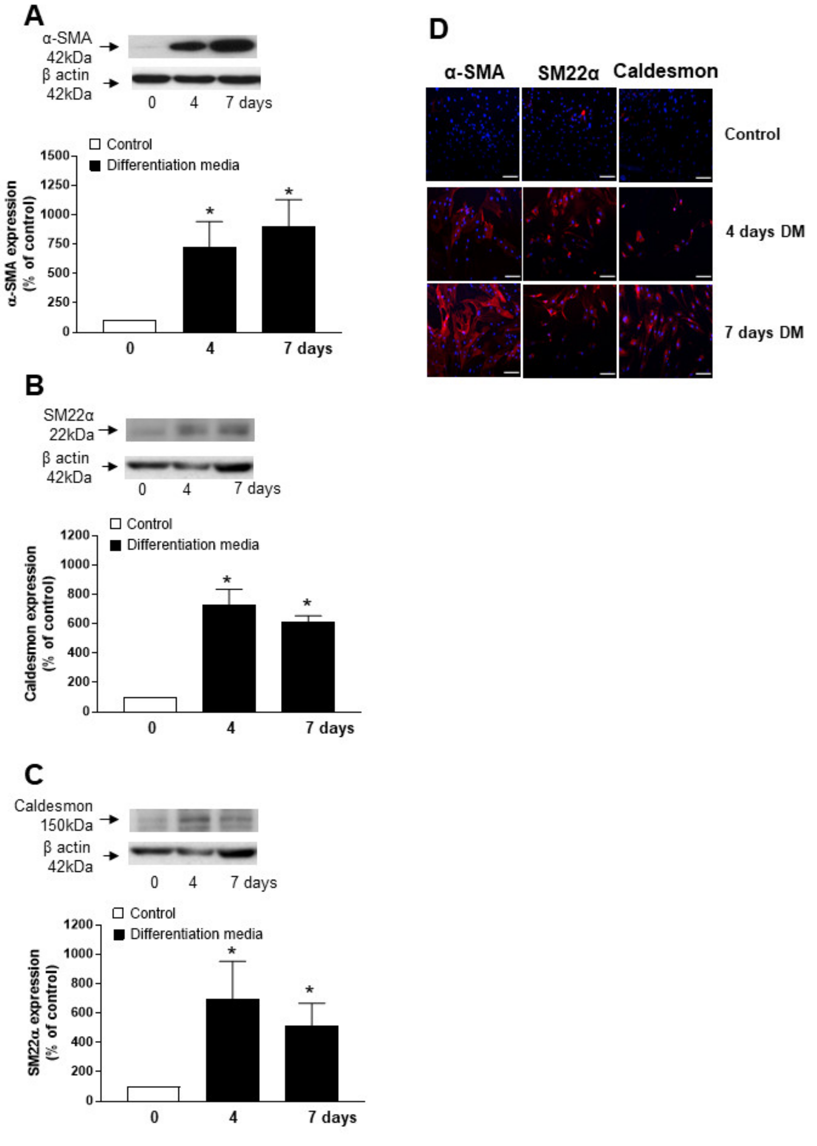

Supplemental Figure S1. TGF-β and BMP-4 induce the differentiation of porcine ASC into SMC. Top are representative immunoblots of the differentiation effects of TGF-β and BMP-4 on αSMA (**A**), SM22α (**B**) and caldesmon (**C**) expression. Corresponding bar graph demonstrate the effect (0, 4 and 7 days) effect of TGF-β and BMP-4 on αSMA, Caldesmon and SM22α expression. (C) Fluorescence microscopy was also used to evaluate αSMA, Caldesmon and SM22α in porcine ASC. Scale bar 100 μm. Results are mean ± SEM of 5 experiments. * $p < 0.05$ vs control.

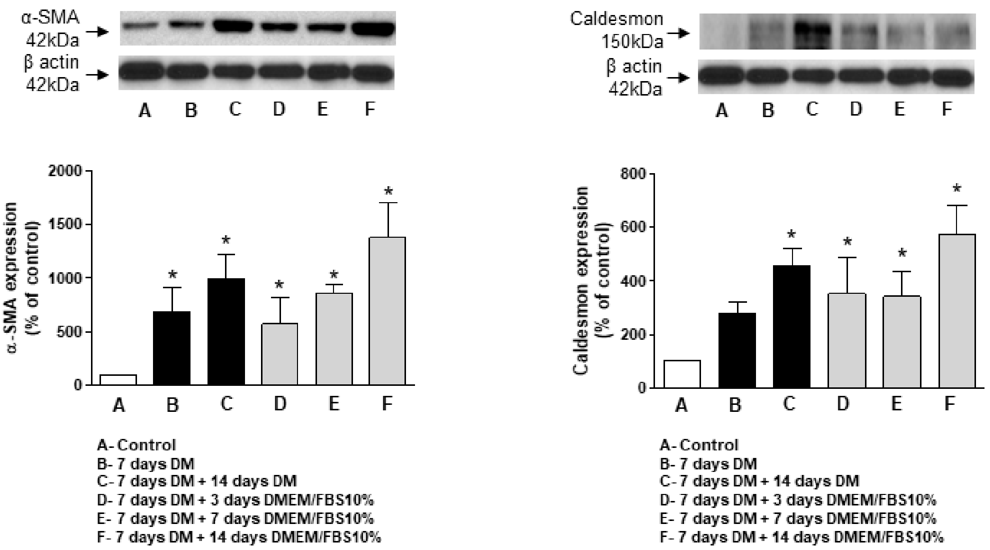

Supplemental Figure S2. TGF-β and BMP-4 induced the differentiation of porcine ASC into SMC is stable for up to 21 days. Top are representative immunoblots for αSMA and Caldesmon. Corresponding bar graph demonstrate the stability of differentiation when cells were differentiated for 7 days and then either maintained in differentiation media containing TGF-β and BMP-4 or transferred to DMEM supplemented with FBS for indicated times. Results are mean ± SEM of 5 experiments. * $p < 0.05$ vs non-differentiated ASC.
